# Time Sequence Deep Learning Model for Ubiquitous Tabular Data with Unique 3D Tensors Manipulation

**DOI:** 10.3390/e26090783

**Published:** 2024-09-12

**Authors:** Adaleta Gicic, Dženana Đonko, Abdulhamit Subasi

**Affiliations:** 1 Faculty of Electrical Engineering, University of Sarajevo, 71000 Sarajevo, Bosnia and Herzegovina; ddonko@etf.unsa.ba; 2 Institute of Biomedicine, Faculty of Medicine, University of Turku, 20520 Turku, Finland; abdulhamit.subasi@utu.fi; 3 Department of Computer Science, College of Engineering, Effat University, Jeddah 21478, Saudi Arabia

**Keywords:** deep learning, deep neural network architectures, Stacked Bidirectional LSTM, time sequence forecasting algorithms, prediction with tabular data, tabular datasets

## Abstract

Although deep learning (DL) algorithms have been proved to be effective in diverse research domains, their application in developing models for tabular data remains limited. Models trained on tabular data demonstrate higher efficacy using traditional machine learning models than DL models, which are largely attributed to the size and structure of tabular datasets and the specific application contexts in which they are utilized. Thus, the primary objective of this paper is to propose a method to use the supremacy of Stacked Bidirectional LSTM (Long Short-Term Memory) deep learning algorithms in pattern discovery incorporating tabular data with customized 3D tensor modeling in feeding neural networks. Our findings are empirically validated using six diverse, publicly available datasets each varying in size and learning objectives. This paper proves that the proposed model based on time-sequence DL algorithms, which were generally described as inadequate when dealing with tabular data, yields satisfactory results and competes effectively with other algorithms specifically designed for tabular data. An additional benefit of this approach is its ability to preserve simplicity while ensuring fast model training also with large datasets. Even with extremely small datasets, models can be applied to achieve exceptional predictive results and fully utilize their capacity.

## 1. Introduction

Deep learning methods excel remarkably for classification or data generation tasks on homogeneous data types such as images, audio, and text. However, deep learning algorithms in predictive modeling with tabular datasets have not yet been effectively and extensively applied [1,2]. One reason for this is attributed to the fact that DL models trained on conventional tabular data may not be as efficient as classical machine learning models since their adaptation to tabular data for data generation tasks remains highly challenging [1]. The second cause for the insufficient application of deep learning techniques in building models based on tabular data is that they cannot reach their full capacity if datasets are of small to medium sizes [3]. Deep learning models need large datasets to perform exceptionally well, but most applications have small or inadequate data to train DL frameworks [4]. This results in a need for manual labeling and human intervention that requires a lot of effort and expertise, and it is also time consuming and prone to errors [4]. Data scarcity presents a significant challenge when training DL models with multi-layered architectures with millions and billions of hidden features. That can result in poor model generalizability and transferability. In cases where the training is conducted on limited datasets, hidden features can become radically underdetermined due to insufficient data [4].

We proposed a model based on time-series deep learning Stacked Bidirectional LSTM network architecture with unique modeling for tabular data. It should be emphasized that the independence of the rows within the sequence is the most important postulate because the inherent nature of the data does not establish interdependence between them, since it is not time-sequence datasets that we apply to time-sequence algorithms.

Additionally, models proposed in previous scientific publications were usually inadequate compared to each other, and existing works often employ varying benchmarks and experimental procedures. Consequently, researchers and practitioners find it unclear which models are the most effective. Furthermore, the field still lacks effective baselines, which should provide competitive performance across different problems [5]. For this reason, we performed a more comprehensive evaluation of the model’s effectiveness using seven metrics: AUC, Accuracy, RMSE, Precision, Recall, F1-score, and Kappa, although most previous works evaluated performance on one or two measures such as Accuracy and AUC.

In this paper, six publicly available tabular datasets are used. Four credit-scoring datasets [6,7,8,9] of various sizes are used to prove that DL algorithms designed for time-series predictions can be effectively utilized using the approach proposed within this paper. Additionally, two different, well-known real-world tabular datasets, the HELOC [10] and Adult Income datasets [11], of different sizes and different learning objectives used in Borisov’s study [1] are also included to prove, extend, and substantiate our claims within the context of tabular data in general.

This paper is organized as follows: This section briefly explains the challenges deep learning models face with tabular data. Section 2 reviews related work in credit scoring and deep learning. Section 3 covers materials and methods, while Section 4 presents the proposed deep learning model with details regarding novelty. Results, discussion, and analysis of the achieved results are stated in Section 5. The concluding remarks and future works are presented in Section 6.

## 2. Background and Related Work

While deep learning has enabled tremendous progress on text and image datasets, its superiority on tabular data is not clear [2]. A detailed review [12] has emphasized the absence of review papers dedicated exclusively to DL for credit scoring, despite the growing interest in developing such models for time-series forecasting. DL algorithms cannot reach their maximum capacity when operating with sparse data, and thus, state-of-the-art algorithms perform better when utilizing a low-complexity model [3]. A comprehensive review from the year 2024 [13] on deep learning and machine learning techniques for credit scoring shows that there is insufficient research centered specifically on the utilization of LSTM-based networks in the field of credit scoring since the implementation of deep learning neural networks is specifically designed for sequential data analysis. Based on the study [13], two papers address this [14,15] with implementation in credit scoring. Research [15] introduced the Word2vec technique for word representation as vectors. The dataset in that research was a real-world dataset P2P lending platform in China but it was not a classical tabular credit scoring dataset. The main reason for this is that DL demands a large amount of data to achieve exceptional performance [4]. However, this is not true in credit-scoring scenarios with small to mid-size datasets.

Another review [16] unlike the previous one [1] that analyzed deep learning with tabular data in general is focused only on deep learning in credit scoring with the utilization of German, Australian, Japanese, and Taiwan datasets. Only one reference within the review [16] utilized the LSTM network [17] with results presented for the German (numerical) dataset [6] but for only one metric that is AUC with a value of 0.803, and metric Accuracy was not shown in the presented results. So, this also confirms the statement that the current utilization of LSTM in credit scoring is insufficient, and benchmarks and experimental procedures are inadequate and poor. However, our previous studies addressed the problem of effectively applying LSTM-based models in credit-scoring modeling [18,19].

## 3. Materials and Methods

### 3.1. Datasets

Six publicly available datasets are used in this paper: four credit-scoring datasets and two of the five most popular datasets from the comprehensive study [1] of application of deep learning models for tabular datasets: the Home Equity Line of Credit (HELOC) dataset [10] and Adult Income dataset [11]. The HELOC dataset with anonymized data from actual homeowners who applied for home equity lines of credit contains information from their credit report to forecast whether they will repay their HELOC account within a two-year timeframe. Another popular dataset is the Adult Income dataset [11] which is among the most popular tabular datasets used in the surveyed work [1]. It encompasses fundamental details about individuals, including age, gender, and level of education with a binary target denoting high or low income. The largest dataset used in this study is the “Give Me Some Credit” dataset [9], which involves predicting the likelihood that an individual will experience financial difficulties within the next two years. Other datasets used in this paper are well-known public credit scoring datasets: German Credit Data [6], Australian Credit Approval [7], and Taiwan Default of Credit Card Clients Dataset [8]. A general overview of the datasets used in this study is given in Table 1.

### 3.2. Preliminaries

In this paper, we propose a model based on Stacked Bidirectional LSTM. Bidirectional LSTM, an extension of the traditional LSTM architecture, consists of numerous memory cells. The concept of two-way LSTM is based on reading training data in two-time directions by training a neural network [20]. In sequential classifications, only the left context is considered, so the aggregate vector is created by reading the sequence of inputs from left to right. 

Formulas (1)–(6) represent the form of the forward pass of the LSTM unit:

*i*_*t*_ = *σ*(*w*_*i*_[*h*_*t*−1_, *x*_*t*_] + *b*_*i*_), (1)

*f*_*t*_ = *σ*(*w*_*f*_[*h*_*t*−1_, *x*_*t*_] + *b*_*f*_), (2)

*o*_*t*_ = *σ*(*w*_*o*_[*h*_*t*−1_, *x*_*t*_] + *b*_*o*_), (3)

(4) $$\tilde{c_{t}}=tanh(w_{c}[h_{t-1},\;x_{t}]+b_{c})$$

(5) $$c_{t}=f_{t}\;*c_{t-1}+i_{t}*\tilde{c_{t}}$$

*h*_*t*_ = *o*_*t*_ ∗ *tanh*(*c*_*t*_), (6)

where *i_t_* stands for input gate, *f_t_* represents the forget gate, *o_t_* represents the output gate, *x_t_* represents the current timestamp, *b_i_* represents the bias vector associated with the input gate, *b_o_* represents the bias vector for the output gate, *b_f_* represents the bias vector specific to the forget gate, *h*_*t*−1_ represents the hidden state from previous time step and input to the LSTM cell at time step t, *h_t_* represents the hidden state from at the current time step and the output of the LSTM cell at time step *t*, *w_i_* represents the weight matrix for the input gate, *w_o_* represents the weight matrix for the output gate, *w_f_* represents the weight matrix for the forget gate, *w_c_* represents the weight matrix for the cell state update, *σ* represents the sigmoid function, *c_t_* represents the cell state, *c*_*t*−1_ represents the cell state from the previous time step, *x_t_* represents the input vector at the current time step, tanh represents the hyperbolic tangent function, and *x* is the element-wise vector/matrix multiplication operator.

It has been proved that bidirectional networks are substantially better than unidirectional ones in many fields [21], and therefore they are applied in our proposed predictive model. At any given point within a sequence, the bidirectional network has complete, sequential information about all points before and after it, enhancing its ability to model temporal dependencies [22].

By stacking multi-layer LSTM networks, the model can capture even more complex patterns in data sequences. The architecture of Stacked Bidirectional LSTM has the output hidden layer that is fed as the input into the subsequent hidden layer and differs from classic Bidirectional LSTM in the number of those hidden layers. The number of layers can vary. Although it is considered that architecture with more hidden layers can result in better performance, it should not have to be the case in practice [19]. Stacked Bidirectional LSTM takes context information by concatenating left and right summary vectors, thus performing better than unidirectional deep neural architecture and solving vanishing gradient problems.

## 4. Proposed Time Sequence Deep Learning Predictive Model for Tabular Data

To establish the validity of the proposed concept and to prove the hypothesis that the application of time sequence deep learning forecasting in predictive modeling with tabular data is viable, the Stacked Bidirectional LSTM algorithm is deployed together with a novelty approach regarding the adaptation of 3D tensors.

### 4.1. Novelty in Shifting Perspective for Utilization of Time Sequence DL

The main objective of this paper is to utilize the Stacked Bidirectional LSTM time sequence algorithm to operate optimally with tabular data. “Specificity” is that algorithms based on LSTM networks require the application of tensors for time-series data. The objective can be achieved by shifting the perspective on how algorithms can exploit their possibilities without losing the predictive power by putting the classes (results) of independent loan applicants into a sequence of events because they are not correlated nor connected to other applications by any means, since classes are only dependent on an applicant’s attributes. The behavior of one client is independent of the behavior of other unrelated clients over time. In this methodology, emphasis is placed solely on the characteristics relating to the observed client, finding patterns in that context. Accordingly, characteristics (features) are treated as the last value in the sequence of events to be considered.

One of the key parts of creating our deep learning model is choosing an adequate 3D tensor depending on the applied dataset. It is necessary to define tensor dimensions that match: batch size, time steps, and features. Thus, we must move the perception on how patterns are learned by algorithm and how data are fed into neural networks.

The main idea is founded on the usage of sliding window chunks that do not need to be shaped to take past events into account due to the nature of tabular datasets. Instead, a fixed sliding approach is taken without overlapping the time-series data where the features themselves are treated as time-series data and the next value to predict is the class itself.

**Figure 1:**
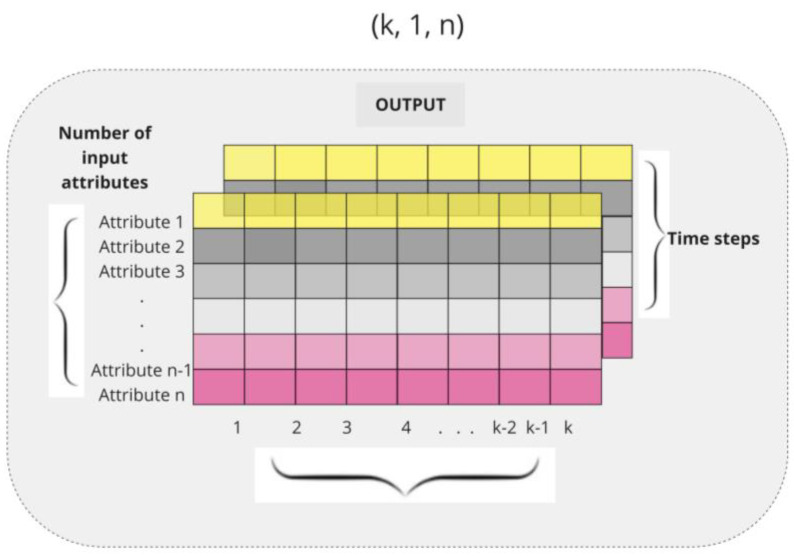
Shaping of 3D tensors.

An input tensor is created by reshaping the scaled feature tensor to a 3D tensor where the first dimension represents the number of samples, the second dimension represents the time steps, which is 1, since no sliding window approach is applied, and the third dimension represents the features. So, the proposed shape of the 3D tensor is (number_of_samples, 1, number_of_features), which is applied independently for all datasets used in this study. The target vector Y does not undergo any reshaping and remains a 1D vector shaped (number_of_samples) (Figure 1). Thus, each sample is a tensor with a single row representing one time step. The LSTM model in this application receives input with a one time step per sample.

This approach simplifies the input data and reduces the computational complexity of the model. At the same time, it will not deteriorate performance, which would be the case in time sequence problems. Additionally, it cannot limit the model’s ability to capture long-term dependencies in the data since events are not interdependent.

The standard approach would imply the construction of an input tensor using a sliding window approach where each sequence of x time steps presents the number of input nodes. On the contrary, classical models are designed to look back at x time steps with the same target vector. Due to the larger input tensor, the standard approach increases both the time needed for model training and the complexity of the model. However, using our approach, the time for model training and the complexity of the model are both decreased.

### 4.2. Model Architecture

**Figure 2:**
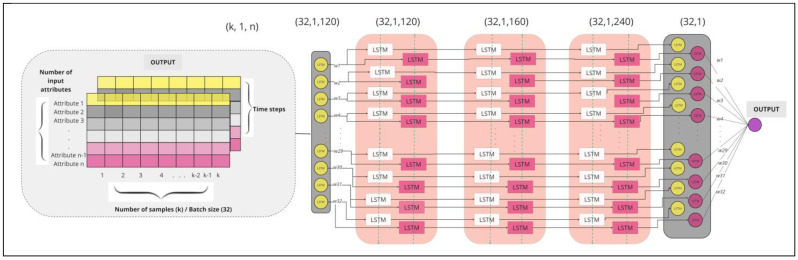
The architecture of Stacked Bidirectional LSTM model with 3D tensor.

In this research, we have introduced a model that combines and leverages the advantages of Stacked Bidirectional LSTM architecture. Figure 2 shows that each data input is a three-dimensional array with the number of samples as one dimension, the time step of one as the second dimension, and the number of characteristics as the third dimension. Shaped this way, data are fed to the neural network which consists of one input layer, three hidden layers, and one dense layer. The number of nodes in each layer and values of hyperparameters of the proposed network are given in Table 2.

By increasing the number of nodes toward the deeper layers, the model’s capacity to learn more complex patterns also increases, refining them and improving the overall performance. The last hidden layer is connected to the dense layer where each neuron receives input from all the neurons from the previous layer (Figure 2). The dense layer has two possible output values, 0 or 1, representing the prediction result.

The proposed model consists of three hidden layers and is trained using the hyperparameters outlined in Table 2.

The model includes dropout layers after each bidirectional LSTM layer to prevent overfitting with dropout rates of 0.2 together with different numbers of units within the layers. Adam’s optimization method and RMSE metrics minimize mean square error (MSE). The Stacked Bidirectional LSTM network consists of two LSTMs; thus, it requires approximately double the training time compared to a single LSTM. Additionally, the training times of multi-layer models are nearly linearly proportional to the number of layers. Due to the unique data shaping described in Section 4.1, the model has efficient training speed. Each model is trained on all six publicly available tabular datasets tested and compared with results from previous publications. Data are first shuffled to ensure randomness. As a scaling technique, MinMaxScaler was used to make the values of parameters homogeneous (in the range from 0 to 1) and to belong to the same scope to avoid intensive calculations by models. Results are based on standard 5-fold cross-validation and can be found in Section 5.

### 4.3. Development Approach and Code Specifications

The deep learning model was implemented, deployed, and experimented using Python. Algorithm 1 presents the entire process of developing the Stacked Bidirectional LSTM model, encompassing all necessary phases, using pseudo code. Keras as a high-level API was used to streamline the process of building and training neural networks with integrated TensorFlow as the backend for optimized computations and scalability. The pseudocode for the proposed model is provided below (Algorithm 1).
**Algorithm 1.** Time sequence model based on Stacked Bidirectional LSTM networks for tabular datasets.Input: Tabular datasets with x features. Output: Target variable Y is a binary label.# Step 1: Load data  1.1 Loading tabular datasets # Step 2: Preprocess of datasets  2.1 Feature Extraction from Raw Data  2.2 Missing Values Handling  2.3 Vectorization  2.4 Parameter Scaling (normalization with MinMaxScaler; feature range from 0 to 1)  2.5 Encoding  2.6 Outlier Detection  2.7 Optional: Dimensionality Reduction Techniques (feature selection)  2.8 Shuffling preprocessed data to introduce randomness and prevent model bias # Step 3: 3D tensors reshaping  3.1 Transforming input data into 3D tensors as an adequate input to the LSTM-based model.
  • 3D tensor is shaped as (number_of_samples, 1, number_of_features)   • The target vector Y does not undergo any reshaping and remains a 1D vector-shaped (number_of_samples,)  3.2 Defining the input form based on the number of time steps and the number of features  3.3 Separation of data into a training set and test set   3.4 Defining the ratio of data allocated to each set # Step 4: Design, model training, and hyperparameter tuning of Stacked Bidirectional LSTM classifiers  4.1 Initialization of deep learning Stacked Bidirectional LSTM model architecture with hidden layers  4.2 Setting hyperparameters  4.3 Compiling the model with the appropriate loss function, optimizer, and hyperparameters  4.4 Training the model using training data  • Setting the number of epochs and the size of the training series  • Computation of RMSE, loss and accuracy convergence monitoring  4.5 Model Performance Evaluation.     Testing and thresholding # Step 5: Visualization of the results 5.1 Graphical representation of actual values compared to those predicted to visualize the performance of the model 5.2 Analysis of the obtained values to assess the accuracy of the prediction

## 5. Results and Discussion

In this section, validation of the proposed three-layer stacked LSTM model was completed on six popular publicly available tabular datasets. That adds an essential layer of credibility to the research findings, which are transparent and reproducible and can be compared to other studies. Datasets vary in size, feature types, and distributions and are explained in detail in Section 3.1. The total number of different datasets used in this study is six. However, there are seven columns for datasets in Table 3. because German datasets consist of two versions: one with numerical features and the other with categorical features.

A standard 5-fold cross-validation was applied to evaluate a model’s performance, better understand how well it generalizes to an independent dataset, and to ensure robust and reliable performance metrics.

The results in Table 3 show that the proposed deep learning model achieved competitive results. Reproducible results assess the generalizability of the model across different contexts. Seven metrics are used in this study to evaluate model performance: AUC, Accuracy, RMSE, Precision, Recall, F-measure, and Kappa metrics. However, most previous research on this topic benchmarks performance using only Accuracy and AUC [1,16].

The results shown in Table 3 demonstrate better model performance for the German dataset with categorical than with numerical values. This is, however, also the case with models based on classical machine learning algorithms and statistical methods published in other relevant studies. 

Top results are marked in bold, and Australian datasets performed the best, considering most of the measures, which is contrary to the prevalent opinion and scientific evidence [3] that small datasets are not suitable for use with deep learning algorithms. The achieved results even exceeded those published in the paper [14] not only compared to the LSTM-based model proposed but also compared to all other models benchmarked in the mentioned study [14], which is presented in Table 4.

If we compare our results on German (numerical) datasets with the results published in the review [16], it can be concluded that our results surpassed the performance of models analyzed in that review. Additionally, results published in a study [16] related to LSTM are limited, including only AUS with 0.803, while Accuracy was not included. The model proposed in our study achieved an AUC of 0.884 for the German (numerical) dataset.

Comparing the German (categorical) dataset results, we conclude that the accuracy of 88.0% achieved by applying the CNN [16] was better than the accuracy of 87.7% achieved by using the Stacked Bidirectional LSTM model in our study. However, AUC was not reported in that study, thus precluding a direct comparison with our results. Applying the method of a two-stage hybrid default discriminant model based on deep forest accuracy was 81.2%, and the AUC was 0.868 [16].

Performance results presented in the study [16] for the German Credit categorical dataset are presented in Table 5.

Performance results presented in the study [16] for the German Credit numerical dataset are presented in Table 6.

The best results regarding accuracy and RMSE were achieved using the “Give Me Some Credit” dataset. The “Give Me Some Credit” dataset was the largest dataset used in our experiment. Since deep learning algorithms perform best with large datasets, maximizing their performance and effectiveness, this performance was expected.

The Taiwan dataset was applied and examined within the study [23] comparing classical machine learning approaches with the LSTM method. The LSTM-based method outperformed all machine learning techniques with an Accuracy of 0.8233 and an F1-score of 0.4421. Our study, on the other hand, achieved a similar accuracy of 0.8238 and an F1-score of 0.4710 for the Taiwan dataset.

According to the results published in a study [1], for the Adult Income dataset, our results with an accuracy of 73.45% and AUC of 0.8063 were comparable to those achieved using other deep learning techniques. It surpassed the results achieved by MLP, VIME, NAM, TabTransformer, RLN, and STG from the study [1], but it was outperformed by DeepFM, NODE, DeepGBM, Net-DNF, TabNet, and SAINT.

If we compare the results for the Adult Income dataset, our method shows promising results with an accuracy of 85.66% and an AUC of 0.9120.

The results of the performance in a study [1] for the Adult Income and HELOC datasets are presented in Table 7. The methodologies are classified into two categories: Machine Learning and Deep Learning. The evaluation metrics utilized for model assessment include Accuracy and the Area Under the Curve (AUC), with results presented for both datasets

The method proposed in this paper outperformed TabNet, DCN2, SNN, MLP, and ResNet from the study [5]. Although some techniques, such as NODE, AutoInt, GrowNet, and FT-Transformer, showed slightly better performance, the differences were minimal. However, the results presented in the study [5] are based on the ensemble of DL algorithms implemented in a significantly more complex manner than our model. The model proposed in this paper also has efficient training capabilities because it uses smaller input tensors.

The findings displayed in Table 3 challenge the prevailing understanding by demonstrating that the utilization of DL algorithms does not yield satisfactory outcomes for models handling tabular data in general. Moreover, it has been proven to be significantly effective even on small datasets, such as Australian and German datasets when applying the Stacked Bidirectional LSTM algorithm with special data modeling.

There is a constant need to overcome the constraints of different studies that focus on specific aspects of algorithms such as accuracy, interpretability, and computational efficiency with the comparison of different datasets [24]. The results achieved using the proposed model are competitive with those presented in other publications with more complex models. It is important to note that although the training was conducted on the imbalanced dataset, no additional methods were required to balance the data, since LSTM-based models are highly effective in capturing data dependencies.

The comprehensive comparison of model efficiency on different publicly available tabular datasets highlighted the strengths of time sequence deep learning models in the context of tabular datasets. Results are generalizable, and other researchers can replicate the study which enables the verification of our findings.

## 6. Conclusions

A new predictive model was proposed and examined, introducing a novel approach to tabular data using time-sequence forecasting algorithms. Although tabular data still pose a challenge to deep learning models, this paper demonstrates the effectiveness of applying time-series algorithms to ubiquitous tabular data, where rows are independent, neither sequentially related nor time-dependent.

Even though LSTM-based networks are DL networks specifically designed for sequential data analysis and not for tabular data, we have demonstrated in this research how they can be effectively deployed on tabular data even on datasets of small sizes. When working with small datasets, these hidden features can become greatly underdetermined due to limited data, while deep learning models with multi-layered architecture have too many variables.

Regardless of how sophisticated an algorithm is applied, and despite the degree of optimization and fine tuning applied to an algorithm, it most frequently fails to achieve optimal performance or fully utilize its capacity when trained on tabular data.

The improvement is related to the manipulation of datasets, since these types of networks are designed to predict the next value of a time series, whereas our problem is not a time-sequence problem.

We are solving a problem with the imbalanced dataset. However, the results indicated that the introduction of any additional techniques for data balancing was not necessary. However, the complexity of a model can be a limiting factor when the number of samples per class is small. Having small datasets can also be a major problem when training deep learning models with complex layers and numerous hidden features. This can lead to models that do not generalize well or transfer effectively to new situations.

The dimensionality reduction technique was also not applied because the model performance was satisfactory, and that technique could potentially lead to unnecessary model complexity, overfitting, or the deterioration of performance. Thus, an additional benefit of the proposed model lies in the fact that simplicity was also preserved.

Six public datasets played crucial roles in this study, providing a common ground for comparison and validation, allowing researchers to evaluate the ability of the proposed model to generalize well.

## Figures and Tables

**Table 1:** A general overview of the datasets used in this study.

	German	Australian	Taiwan	Give Me Some Credit	HELOC	Adult Income
Samples	1000	690	30,000	150,000	10,459	48,872
Features	20 (cat.) 24 (num)	14	24	11	23	14
Classes	2	2	2	2	2	2
Positive samples	700	307	6636	139,974	5000	11,687
Negative samples	300	383	23,364	10,026	5459	37,155
Imbalance ratio	2.33	1.25	3.52	13.96	1.09	3.18

**Table 2:** A general overview of the Stacked Bidirectional LSTM model hyperparameters.

Hyperparameters	Values	Note
Number of hidden layers	3	
Number of nodes of the input layer	60	Input layer
Number of nodes of 1. hidden layer	60 × 2 = 120	First hidden layer
Number of nodes of 2. hidden layer	80 × 2 = 160	Second hidden layer
Number of nodes of 3. hidden layer	120 × 2 = 240	Third hidden layer
Number of nodes of a dense layer	1	Possible values: 0, 1
Dropout	0.2	
Decay rate	0.97	Default value
Activation function	Relu	
Learning rate	0.01	Default value
Momentum	0.9	
Number of epochs	50	
Batch size	32	
Decision threshold	0.57	

**Table 3:** Stacked Bidirectional LSTM model performance on 6 public datasets.

Measure	German (Categorical)	German (Numerical)	Australian	Taiwan	Give Me Some Credit	HELOC	Adult Income
AUC	0.9232 (±0.0880)	0.8844 (±0.0932)	**0.9519 (±0.0201)**	0.7855 (±0.0066)	0.8316 (±0.0036)	0.8063 (±0.0040)	0.9120 (±0.0033)
Accuracy	0.877 (±0.0676)	0.8720 (±0.0778)	0.8884 (±0.0337)	0.8238 (±0.0060)	**0.9369 (±0.0008)**	0.7345 (±0.0075)	0.8566 (±0.0058)
RMSE	0.2991 (±0.0846)	0.3036 (±0.0914)	0.2848 (±0.0457)	0.3642 (±0.0046)	**0.2249 (±0.0008)**	0.4242 (±0.0030)	0.3136 (±0.0040)
Precision	0.8642 (±0.1093)	0.8260 (±0.1185)	**0.8758 (±0.0226)**	0.7017 (±0.0124)	0.6272 (±0.0402)	0.7296 (±0.0182)	0.7446 (±0.0136)
Recall	0.7077 (±0.1246)	0.7321 (±0.1561)	**0.8743 (±0.0514)**	0.3557 (±0.0318)	0.1520 (±0.0464)	0.7823 (±0.0210)	0.6115 (±0.0383)
F-Measure	0.7769 (±0.1179)	0.7737 (±0.1370)	**0.8745 (±0.0325)**	0.4710 (±0.0261)	0.2395 (±0.0565)	0.7546 (±0.0065)	0.6705 (±0.0207)
Kappa	0.6938 (±0.1629)	0.6859 (±0.1891)	**0.7723 (±0.0672)**	0.3789 (±0.0240)	0.2196 (±0.0517)	0.4663 (±0.0152)	0.5802 (±0.0228)

Note: Bold text indicates the highest results achieved across different datasets, considering all metrics.

**Table 4:** Baseline, ensemble, and LSTM previous model performance on Australian dataset based on [14].

Methods	Accuracy	RMSE	AUC	Precision	Recall	F-Measure	Kappa
DT	86.09	0.3386	0.867	0.861	0.861	0.861	0.7183
SVM	84.64	0.3919	0.853	0.857	0.846	0.847	0.6941
MLP	83.77	0.3763	0.888	0.861	0.843	0.852	0.6722
DT-boosting	84.35	0.3696	0.902	0.853	0.867	0.860	0.6825
SVM-boosting	82.61	0.3459	0.902	0.854	0.828	0.841	0.6493
MLP-boosting	83.77	0.3868	0.856	0.861	0.843	0.852	0.6722
DT-bagging	86.38	0.3215	0.918	0.881	0.872	0.877	0.7245
SVM-bagging	85.22	0.3575	0.893	0.863	0.852	0.853	0.7059
MLP-bagging	85.65	0.3363	0.909	0.876	0.864	0.870	0.71
LSTM	87.22	0.3366	0.914	0.854	0.885	0.869	0.7998
LSTM + GA	89.27%	-	-	-	-	-	-

**Table 5:** Performance credit scoring models for the German (categorical) dataset based on the study [16].

Methods	Accuracy	AUC
IGDFS + GBT classifier	98.66	----
Feature selection + ML classifier selection	93.12	----
NRS + ML ensemble	86.47	----
Hybrid binary particle swarm optimization and gravitational search algorithm (BPSOGSA)	85.78	----
Artificial bee colony-based SVM	84.0	----
Bolasso-based feature selection	84.0	0.713
Fuzzy group decision making (GDM)	82.0	0.824
Heterogeneous ensemble	----	0.684
Ensemble classifiers	----	0.77
Multi-stage ensemble	79.5	0.831

**Table 6:** Performance credit scoring models for the German (numerical) dataset based on the study [16].

Methods	Accuracy	AUC
MCDM-based evaluation approach	------	0.961
DNN (time delay neural network)	88.24	------
GFSS	87.6	0.813
ELM + novel activation function	80.57	0.862
Hybrid approach based on filter approach and multiple population GA	78.53	------
ML + expert knowledge with GA	------	0.789
Step-wise multi-grained augmented boosting DT	77.15	0.792

**Table 7:** Performance credit scoring models for the Adult Income and HELOC datasets based on [1].

		HELOC		Adult	
	Method	Acc	AUC	Acc	AUC
Machine Learning	Linear Model	73.0 ± 0.0	80.1 ± 0.1	82.5 ± 0.2	85.4 ± 0.2
	KNN	72.2 ± 0.0	79.0 ± 0.1	83.2 ± 0.2	87.5 ± 0.2
	Decision Trees	80.3 ± 0.0	89.3 ± 0.1	85.3 ± 0.2	89.8 ± 0.1
	Random Forest	82.1 ± 0.2	90.0 ± 0.2	86.1 ± 0.2	91.7 ± 0.2
	XGBoost	83.5 ± 0.2	92.2 ± 0.0	87.3 ± 0.2	92.8 ± 0.1
	LightGBM	83.5 ± 0.1	92.3 ± 0.0	87.4 ± 0.2	92.9 ± 0· 1
	CatBoost	83.6 ± 0.3	92.4 ± 0.1	87.2 ± 0.2	92.8 ± 0.1
	Model Trees	82.6 ± 0.2	91.5 ± 0.0	85.0 ± 0.2	90.4 ± 0.1
Deep Learning	MLP	73.2 ± 0.3	80.3 ± 0.1	84.8 ± 0.1	90.3 ± 0.2
	VIME	72.7 ± 0.0	79.2 ± 0.0	84.8 ± 0.2	90.5 ± 0.2
	DeepFM	73.6 ± 0.2	80.4 ± 0.1	86.1 ± 0.2	91.7 ± 0.1
	DeepGBM	78.0 ± 0.4	84.1 ± 0.1	84.6 ± 0.3	90.8 ± 0.1
	NODE	79.8 ± 0.2	87.5 ± 0.2	85.6 ± 0.3	91.1 ± 02
	NAM	73.3 ± 0.1	80.7 ± 0.3	83.4 ± 0.1	86.6 ± 0.1
	Net-DNF	82.6 ± 0.4	91.5 ± 0.2	85.7 ± 0.2	91.3 ± 0.1
	TabNet	81.0 ± 0.1	90.0 ± 0.1	85.4 ± 0.2	91.1 ± 0.1
	TabTransformer	73.3 ± 0.1	80, 1 ± 0.2	85.2 ± 0.2	90.6 ± 0.2
	SAINT	82.1 ± 0.3	90.7 ± 0.2	86.1 ± 0.3	91.6 ± 0.2
	RLN	73.2 ± 0.4	80.1 ± 0.4	81.0 ± 1.6	75.9 ± 8.2
	STG	73.1 ± 0.1	80.0 ± 0.1	85.4 ± 0.1	90.9 ± 0.1

## Data Availability

The original data presented in the study are openly available German datasets (categorical and numerical): in the [UCI Machine Learning Repository] repository at [https://archive.ics.uci.edu/dataset/144/statlog+german+credit+data] URL (accessed on May 2024). Australian dataset: in the [UCI Machine Learning Repository] repository at [https://archive.ics.uci.edu/ml/datasets/Statlog+%28Australian+Credit+Approval%29] URL (accessed on May 2024). Taiwan dataset: in the [UCI Machine Learning Repository] at [https://archive.ics.uci.edu/dataset/350/default+of+credit+card+clients] URL (accessed on May 2024). “Give me Some Credit” dataset: in the [kaggle] at [Give me Some Credit] at [https://www.kaggle.com/brycecf/give-me-some-credit-dataset] URL (accessed on May 2024), Home Equity Line of Credit (HELOC) in the [FICO] at [https://community.fico.com/s/explainable-machine-learning-challenge] URL (accessed on May 2024), Adult Income dataset in the [UCI Machine Learning Repository] at [http://archive.ics.uci.edu/dataset/2/adult] URL (accessed on May 2024).

## References

[1] Borisov V., Leemann T., Seßler K., Haug J., Pawelczyk M., Kasneci G. (2022). Deep Neural Networks and Tabular Data: A Survey. IEEE Trans. Neural Netw. Learn. Syst..

[2] Grinsztajn L., Oyallon E., Varoquaux G. (2022). Why do tree-based models still outperform deep learning on tabular data?. arXiv.

[3] Brigato L., Iocchi L. A Close Look at Deep Learning with Small Data. Proceedings of the 25th International Conference on Pattern Recognition (ICPR).

[4] Alzubaidi L., Bai J., Al-Sabaawi A., Santamaría J., Albahri A.S., Al-dabbagh B.S.N., Fadhel M.A., Manoufali M., Zhang J., Al-Timemy A.H. (2023). A survey on deep learning tools dealing with data scarcity: Defnitions, challenges, solutions, tips, and applications. J. Big Data.

[5] Gorishniy Y., Rubachev I., Khrulkov V., Babenko A. (2023). Revisiting Deep Learning Models for Tabular Data. arXiv.

[6] Hofmann H. (1994). UCI Machine Learning Repository: Statlog (German Credit Data) Data Set. Institut fur Statistik und “Okonometrie Universit” at Hamburg. https://archive.ics.uci.edu/dataset/144/statlog+german+credit+dataURL.

[7] Quinlan R. UCI Machine Learning Repository—Statlog (Australian Credit Approval) Dataset. https://archive.ics.uci.edu/ml/datasets/Statlog+%28Australian+Credit+Approval%29URL.

[8] I-Cheng Y. (2016). Default of Credit Card Clients. https://archive.ics.uci.edu/dataset/350/default+of+credit+card+clientsURL.

[9] Freshcorn B. (2011). Give Me Some Credit: 2011 Competition Data. https://www.kaggle.com/brycecf/give-me-some-credit-datasetURL.

[10] FICO (2019). Home Equity Line of Credit (HELOC). https://community.fico.com/s/explainable-machine-learning-challengeURL.

[11] Becker B., Kohavi R. (1996). UCI Machine Learning Repository Adult Dataset. http://archive.ics.uci.edu/dataset/2/adult.

[12] Sezer O.B., Gudelek M.U., Ozbayoglu A.M. (2020). Financial time series forecasting with deep learning: A systematic literature review: 2005–2019. Appl. Soft Comput..

[13] Wube H.D., Esubalew S.Z., Weldesellasie F.F., TDebelee G. (2024). Deep Learning and Machine Learning Techniques for Credit Scoring: A Review. Proceedings of the Pan-African Conference on Artificial Intelligence.

[14] Adisa J., Ojo S., Owolawi P., Pretorius A., Ojo S.O. Credit Score Prediction using Genetic Algorithm-LSTM Technique. Proceedings of the 2022 Conference on Information Communications Technology and Society (ICTAS).

[15] Wang C., Han D., Liu Q., Luo S. (2018). A deep learning approach for credit scoring of peer-to-peer lending using attention mechanism LSTM. IEEE Access.

[16] Hayashi Y. (2022). Emerging Trends in Deep Learning for Credit Scoring: A Review. Electronics.

[17] Shen F., Zhao X., Kou G., Alsaadi F.E. (2020). A new deep learning ensemble credit risk evaluation model with an improved synthetic minority oversampling technique. Appl. Soft Comput..

[18] Gicić A., Đonko D., Subasi A. (2023). Intelligent credit scoring using deep learning methods. Concurr. Comput..

[19] Gicić A., Ðonko D. Proposal of a model for credit risk prediction based on deep learning methods and SMOTE techniques for imbalanced dataset. Proceedings of the 2023 XXIX International Conference on Information, Communication and Automation Technologies (ICAT).

[20] Onan A., Toçoglu M. (2021). A term weighted neural language model and stacked bidirectional LSTM based framework for sarcasm identification. IEEE Access.

[21] Graves A., Jaitly N., Mohamed A.-R. Hybrid speech recognition with deep. Proceedings of the 2013 IEEE Workshop on Automatic Speech Recognition and Understanding (ASRU) IEEE.

[22] Zhang S., Zheng D., Hu X., Yang M. Bidirectional Long Short-Term Memory Networks for Relation Classification. Proceedings of the 29th Pacific Asia Conference on Language, Information and Computation.

[23] Liu R. (2018). Machine Learning Approaches to Predict Default of Credit Card Clients. Mod. Econ..

[24] Lessmann S., Baesens B., Seow H.-V., Thomas L.C. (2015). Benchmarking state-of-the-art classification algorithms for credit scoring: An update of research. Eur. J. Oper. Res..

